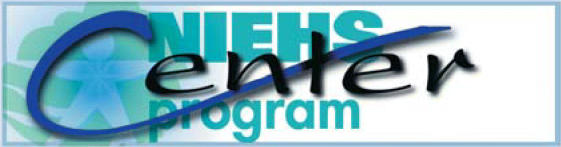# The Environmental Health Science Core Centers Turn Toward Public Health Questions

**Published:** 2007-02

Few NIEHS programs can lay claim to carrying out its director’s stated mission as closely as the Environmental Health Science Core Centers (EHS CCs), which support centralized resources and facilities shared by investigators with existing research projects, thus enhancing productivity and integrating research but—with the exception of pilot project “seed money”—do not add funds to existing projects.

Since its inception in the 1970s, the EHS CC program has grown to 25 centers. Although many scientific and public health advances have been accomplished, the NIEHS is reengineering the EHS CCs to focus on their strengths to support research on human health and disease.

To synergize with the recently released NIEHS Strategic Plan, the next generation of EHS CCs will identify opportunities to translate environmental health research and basic science to public health and the clinic. Chief among the new goals is to develop collaborations and resources that support the integration of basic sciences with clinical research, as well as public health studies of highly exposed populations around the globe.

Each center now will be required to have an Integrative Health Sciences Facility Core (IHSFC), which will facilitate the translation of bench research into public health applications. Efforts will be made to help basic researchers maneuver through the clinical research requirements and will introduce cutting-edge technologies to human studies.

In addition, the EHS CCs will be more active in fostering the careers of young investigators and in drawing needed expertise to the field of environmental health sciences. Training and mentoring junior faculty in environmental health sciences and promoting interactions with established investigators in related disciplines will help young scientists build careers in environmental medicine and become the future scientific leaders.

“As I see it, there are four major areas that will have the greatest impact on preventing disease and improving human health: basic science, disease-oriented research, global environmental health, and of course training tomorrow’s scientists.”

David Schwartz, M.D.

Remarks from the Welcoming Ceremony as NIEHS Director, 24 June 2005

The EHS CCs continue to play a leading role in interacting with local communities, health care professionals, and policy makers. Although the Community Outreach and Education Core (COEC) is now optional, it remains an important component in the EHS CCs. Outreach to the community and health professions has been of high quality, especially distinguished by heroic efforts following Hurricanes Katrina and Rita, and has played an essential role in studies of the health consequences of diesel-related air pollution in the Los Angeles port region. NIEHS remains committed to COEC, and an incentive of $100,000 is provided to centers that pursue these aims.

The EHS CCs have provided a solid backbone of environmental health research in the past and will continue to promote collaborations and initiatives that are essential to attaining the NIEHS vision of preventing disease and improving health through practical applications of novel understanding of environmental sciences. The EHS CCs will continue to evolve as they develop emerging opportunities, attract top researchers, lead in clinical application of those technologies, and foster collaborations that will be essential for the exciting advances of the next century.

Contacts

**Leslie Reinlib, Ph.D.** | reinlib@niehs.nih.gov

**Gwen Collman, Ph.D.** | collman@niehs.nih.gov

**Liam O’Fallon** | ofallon@niehs.nih.gov

## Figures and Tables

**Figure:**